# A novel approach for breast cancer detection using a Nesterov accelerated adam optimizer with an attention mechanism

**DOI:** 10.1038/s41598-025-12070-y

**Published:** 2025-07-25

**Authors:** Abeer Saber, Tamer Emara, Samar Elbedwehy, Esraa Hassan

**Affiliations:** 1 Information Technology Department, Faculty of Computers and Artificial Intelligence, Damietta University, Damietta, 34517 Egypt; 2 Department of Data Science, Faculty of Artifcial Intelligence, Kafrelsheikh University, Kafrelsheikh, 33511 Egypt; 3 Faculty of Artificial Intelligence, Kafrelsheikh University, Kafrelsheikh, 33511 Egypt

**Keywords:** Breast cancer, Deep learning, Image processing, Augmentation, Optimization, Computational biology and bioinformatics, Machine learning

## Abstract

Image-based automatic breast tumor detection has become a significant research focus, driven by recent advancements in machine learning (ML) algorithms. Traditional disease detection methods often involve manual feature extraction from images, a process requiring extensive expertise from specialists and pathologists. This labor-intensive approach is not only time-consuming but also impractical for widespread application. However, advancements in digital technologies and computer vision have enabled convolutional neural networks (CNNs) to learn features automatically, thereby overcoming these challenges. This paper presents a deep neural network model based on the MobileNet-V2 architecture, enhanced with a convolutional block attention mechanism for identifying tumor types in ultrasound images. The attention module improves the MobileNet-V2 model’s performance by highlighting disease-affected areas within the images. The proposed model refines features extracted by MobileNet-V2 using the Nesterov-accelerated Adaptive Moment Estimation (Nadam) optimizer. This integration enhances convergence and stability, leading to improved classification accuracy. The proposed approach was evaluated on the BUSI ultrasound image dataset. Experimental results demonstrated strong performance, achieving an accuracy of 99.1%, sensitivity of 99.7%, specificity of 99.5%, precision of 97.7%, and an area under the curve (AUC) of 1.0 using an 80–20 data split. Additionally, under 10-fold cross-validation, the model achieved an accuracy of 98.7%, sensitivity of 99.1%, specificity of 98.3%, precision of 98.4%, F1-score of 98.04%, and an AUC of 0.99.

## Introduction

**Fig. 1:**
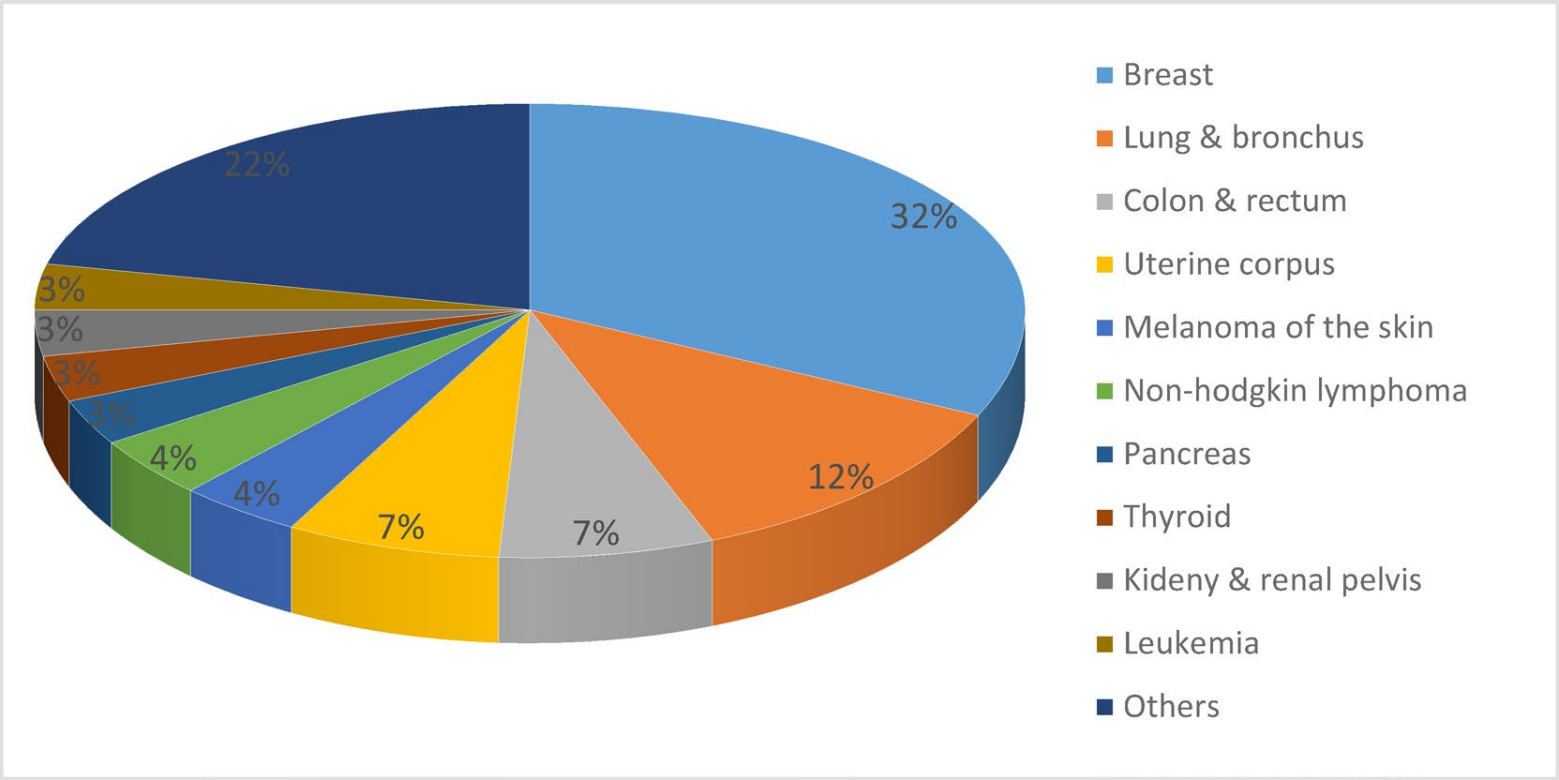
The new cancer case estimation in female [2024].

Cancer is a collection of diseases characterized by the uncontrolled proliferation of abnormal cells. These cells can form malignant tumors that invade and displace healthy tissue. Cancer has represented a significant health burden throughout history and remains a major concern globally. Figure 1 illustrates the estimated number of recent cancers diagnoses in women in the United States for the year 2024. As can be observed, breast, lung, and colorectal cancers are the most prevalent types diagnosed in women, collectively accounting for 51% of all cases. Notably, breast cancer (BC) is the most common cancer among women, representing 32% of all diagnoses^1^.

BC is a category of cancer that predominantly affects women. It is characterized by the uncontrolled proliferation of abnormal breast cells, often leading to the formation of a lump or tumor. These lesions can be benign, exhibiting slow growth and remaining localized, or malignant, demonstrating rapid growth and the potential to spread to nearby tissues. BCs are generally categorized as lobular, originating in the milk-producing lobules, or ductal, originating in the milk-carrying ducts. Several factors have been associated with an increased risk of BC, including modifiable factors such as physical inactivity and obesity, as well as non-modifiable factors like genetics, early menstruation, and late menopause^2,3^.

Early detection and classification of BC are crucial for improving patient outcomes, given that it remains a leading cause of death among women^4^. Timely diagnosis allows for more effective treatment interventions, with studies indicating that 90% of patients with BC identified at an early stage achieve a cure. This significant potential for survival has motivated researchers to develop more accurate frameworks for BC detection.

Imaging modalities are particularly valuable for identifying breast abnormalities due to the superficial location of the breasts. Mammograms and ultrasounds are commonly employed imaging techniques for the prompt identification of BC. Mammography utilizes low-dose X-rays to obtain images of the breast from various angles, aiding in diagnosis. In contrast, ultrasounds are non-radioactive and minimally invasive procedures that use sound waves to produce images of the breast from different perspectives, typically involving a probe that applies gentle pressure. Breast ultrasound, as a non-invasive imaging method employing sound waves to generate images of the breast’s interior, is frequently used in conjunction with mammograms, particularly for women with dense breast tissue or when a suspicious area is identified on a mammogram^5^.

Traditional manual disease diagnosis often suffers from high costs, lengthy procedures, and a potential for errors. Early-stage symptoms of abnormalities can be subtle and easily overlooked, making manual detection a challenging task. To address these limitations, computer-aided detection (CAD) methods have emerged as valuable tools for automated BC detection. By assisting radiologists, these systems offer a more reliable, efficient, and cost-effective diagnostic approach.

CNNs are a powerful type of DL model capable of learning directly from input data, eliminating the need for manual feature extraction. They are particularly effective for tasks such as object, person, and scene detection, excelling at recognizing patterns within images. CNNs demonstrate impressive generalizability across various recognition tasks and are specifically designed to leverage the two-dimensional nature of images, learning to differentiate among various aspects of the input. However, training deep CNNs typically requires a substantial amount of data, which can be a limitation in the medical field, especially for BC^6–14^.

**Fig. 2:**
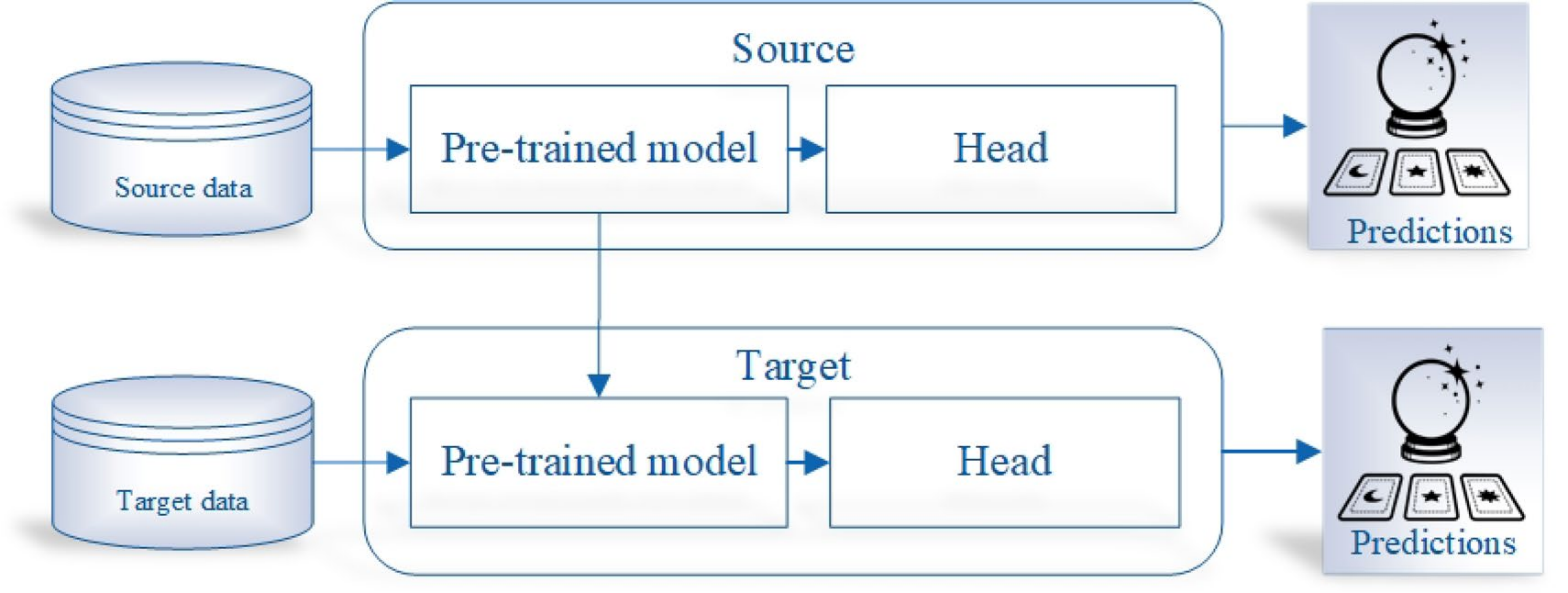
The process of transfer learning.

Transfer learning (TL) is a widely adopted technique in DL applications and has proven particularly valuable in the medical domain, where datasets are often limited. TL aims to improve learning on a target task by transferring knowledge gained from a related source task^15^. Figure 2 illustrates the process of TL.

This paper introduces a deep neural network model based on the MobileNet-V2 architecture, enhanced with a convolutional block attention mechanism to improve the identification of tumor types in ultrasound images. The attention module enhances the model’s performance by focusing on disease-affected regions within the images. Furthermore, the proposed model refines the features extracted by MobileNet-V2 through the application of the Nadam optimization algorithm.

The key contributions of this paper can be summarized as follows:

1. An enhanced CNN model, leveraging the Nadam optimizer and incorporating attention mechanisms, is presented to improve diagnostic accuracy by focusing on relevant image features.
2. The integration of MobileNet-V2 with an attention module offers potential benefits such as improved performance and reduced computational cost.
3. An attention module is strategically added after the final convolutional layer to help the model focus on the most important features in the resulting feature map.
4. The performance of the proposed model is rigorously assessed using standard evaluation metrics, including precision, sensitivity, specificity, and accuracy.

The structure of this paper is as follows: “Related works” section provides a review of current research in the field. “Proposed methodology” section details our proposed model for detecting and classifying BC using enhanced transfer learning techniques. “Experimental results” section presents the experimental analysis of our model’s performance on real ultrasound data. Finally, “Conclusion and future work” section concludes the presented work.

## Related works

Early detection of BC is crucial for improving patient outcomes, as it remains a major cause of cancer-related fatalities among women worldwide. Deep learning (DL) models offer a promising approach for detecting and classifying BC in mammography and histopathological images. Research has demonstrated the effectiveness of various DL techniques, including attention-based models, convolutional neural networks (CNNs) incorporating small Squeeze-and-Excitation (SE) blocks, and multi-task learning paradigms. Additionally, deep CNNs have shown exceptional accuracy in classifying BC.

Sahu et al.^16^ developed a DL -based ensemble classifier for breast cancer detection. This approach combines three transfer learning models and utilizes the Laplacian of Gaussian along with modified high-boosting techniques. The method demonstrates impressive classification performance, achieving an accuracy of 99.17% on the mini-DDSM dataset and 97.75% on the BUSI dataset. It is versatile and reliable, making it suitable for multimodal datasets^1^.

Alnowaiser et al.^17^ developed a methodology that employs CNNs like DenseNet-121 and VGG-16 for feature extraction, with bidirectional long short-term memory (LSTM) layers for temporal feature extraction. The model’s performance, evaluated on the Mammographic Image Analysis Society (MIAS) and INbreast datasets, is impressive.

Saber et al.^18^ proposed a DL model capable of detecting and identifying breast masses by integrating transfer learning (TL) and long short-term memory techniques. This model employs an 80 − 20 split for training and testing and leverages pre-trained networks such as SqueezeNet and DenseNet to extract relevant features. The model’s performance is evaluated using metrics like accuracy, sensitivity, specificity, precision, and the area under the receiver operating characteristic (ROC) curve. The results effectively showcase the model’s capability in accurately detecting breast tumors.

Ahmed et al.^19^ proposed a model that enhances breast image quality and reduces computational load through preprocessing techniques. By transferring learned parameters, the model improves its classification performance. The model’s effectiveness was assessed using four metrics: accuracy, sensitivity, specificity, and the area under the curve (AUC), achieving impressive scores of 97.1%, 96.3%, 97.9%, and 0.988%, respectively.

Saber et al.^20^ proposed a novel DL model for automatically detecting and classifying breast tumors by employing TL techniques. The model leverages pre-trained networks, including VGG-19, VGG16, and InceptionV3, to expedite training and enhance classification accuracy. The Mammographic Image Analysis Society dataset serves as the source for feature extraction. The model demonstrates superior performance compared to VGG19 and Inception V3 in terms of accuracy, sensitivity, and specificity.

Saber et al.^21^ introduced a DL model that utilizes TL techniques to automatically detect and diagnose potential BC regions within mammograms. The model employs an 80 − 20 split and cross-validation to effectively train and evaluate the model, extracting features from a mammographic image analysis dataset using pre-trained CNN architectures. The model achieves the best performance compared with other models in terms of accuracy, sensitivity, specificity, precision, F-score, and AUC, with results ranging from 98.96 to 0.995%.

Tagnamas et al.^22^ developed a two-encoder model that employs EfficientNetV2 and a modified Vision Transformer (ViT) encoder to identify tumor regions in the BUSI dataset. The self-attention mechanism of the ViT encoder captures global features, while the EfficientNetV2 encoder preserves local details. A Channel Attention Fusion module fuses the extracted features, resulting in segmentation maps. The method classifies tumors into benign and malignant using MLP-Mixer, outperforming previous work.

Ali et al.^23^ proposed a model that uses an ensemble technique, transfer learning (TL), and data augmentation to optimize its learning process and adapt to new datasets. It leverages pre-trained models like Inception, ResNet50, and DenseNet121 for feature extraction from the BUSI dataset. Data augmentation generates new training images, increasing dataset diversity. The model’s effectiveness is evaluated based on accuracy scores, consistently achieving a 90% accuracy rate for both benign and malignant images.

Mishra et al.^24^ proposed a machine learning (ML)-radiomics classification pipeline for efficient predictive modelling in medical imaging datasets. It considers multiple images features, eliminates redundant features, and uses synthetic minority oversampling technique (SMOTE) to address class imbalance. The pipeline trains various ML models on the BUSI dataset and achieves a high accuracy of 0.974.

Mahesh et al.^25^ presented an optimized EfficientNetB7 framework with a targeted augmentation strategy, improving model robustness and generalizability. The model achieves a 98.29% diagnostic accuracy on a test dataset, surpassing benchmarks in breast ultrasound image analysis. This model could revolutionize oncological care by aiding early detection and informed management of BC.

While prior studies have reported high accuracy using CNNs, transfer learning, and ensemble methods, they often suffer from limited generalizability across diverse datasets, high computational complexity due to the use of multiple pre-trained models or transformer-based architectures, and a lack of interpretability, which is critical in clinical decision-making. Additionally, some models rely heavily on preprocessing steps or data augmentation to address class imbalance, potentially affecting robustness. This study presents an enhanced CNN model that integrates MobileNet-V2 with an attention module and leverages the Nadam optimizer to improve diagnostic accuracy while reducing computational cost. By strategically placing the attention mechanism after the final convolutional layer, the model effectively focuses on the most relevant image features. Its performance is thoroughly evaluated using standard metrics such as precision, sensitivity, specificity, and accuracy.

## Proposed methodology

**Fig. 3:**
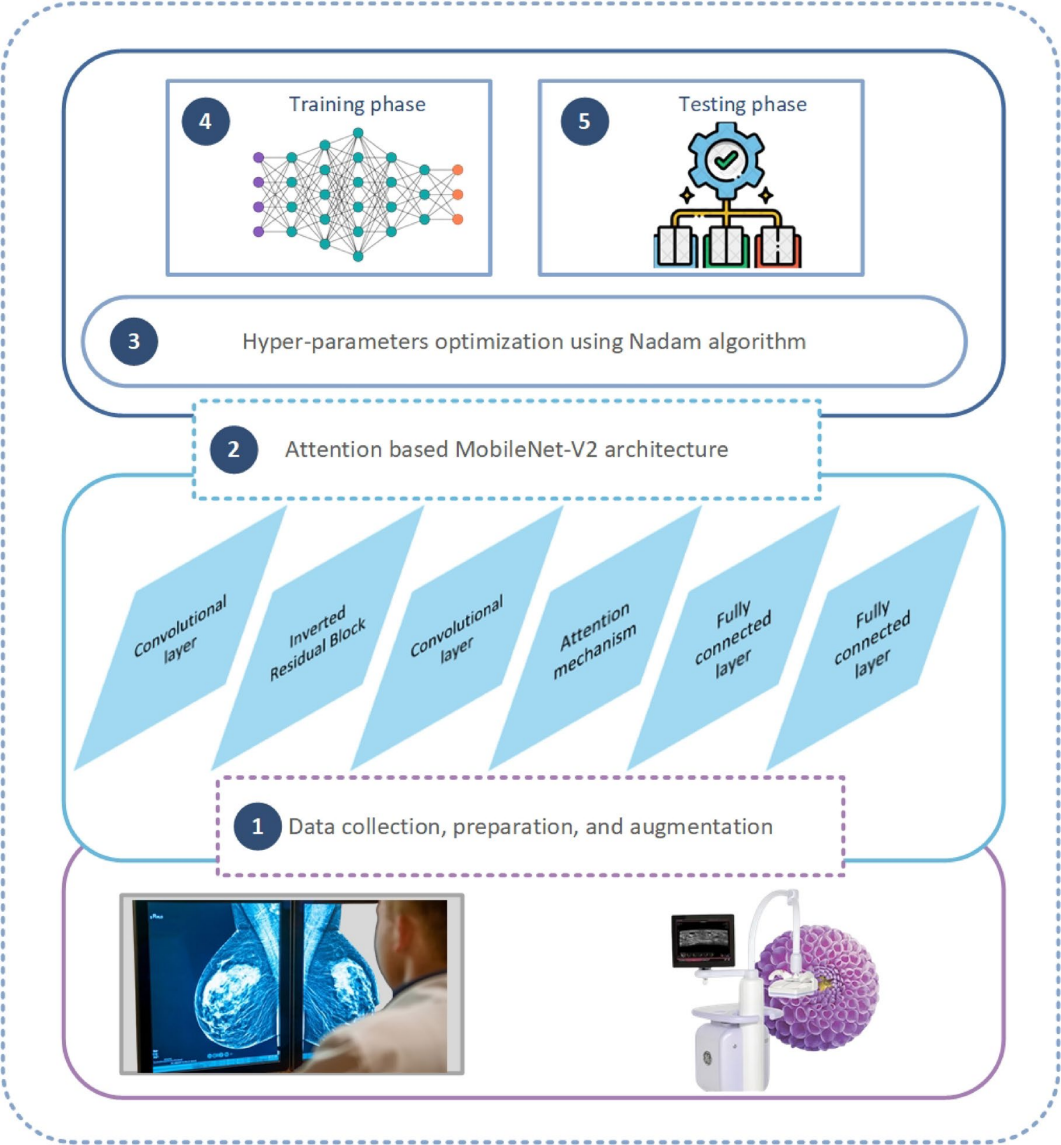
The proposed framework.

The presented method for detecting and classifying BC comprises five major components. These components facilitate data preprocessing, while other components focus on transferring and optimizing CNN parameters, as illustrated in Fig. 3.

### Data collection, preparation, and augmentation

In this paper, several data preprocessing techniques were implemented over the BUSI ultrasound image dataset, which includes breast ultrasound images categorized as normal, benign, and malignant. To enhance the quality of the input data and improve model performance, we applied several preprocessing and augmentation techniques as follows:

1. Noise Reduction:
  Speckle noise commonly affects ultrasound images and can reduce classification accuracy. To mitigate this, we applied a median filter during preprocessing, which smoothed image textures while preserving key anatomical edges necessary for reliable classification.
2. Image Normalization:
  All images were resized to a uniform dimension (224 × 224 pixels) and pixel values were normalized to the range [0, 1] to ensure consistency across the dataset and to improve convergence during model training. This process improves the efficiency of model training by centering the data and aligning it with the expected data distribution.
3. Data Augmentation:
  To mitigate the risk of overfitting and improve model generalization, we applied several data augmentation techniques during training. The augmentation methods applied consisted of rotation, flipping, as well as adjustments in blur, contrast, and sharpening. These methods were crucial for enhancing the dataset diversity and improving the model’s efficiency. By applying these augmentation techniques, we aimed to increase the number of training samples and provide the model with a more balanced representation of all classes. This approach helps to prevent the model from becoming biased towards the majority classes and improves its ability to correctly identify the features of the minority class. We were careful to apply only augmentations that preserve the clinically relevant characteristics of the ultrasound images, avoiding transformations that could introduce artificial or misleading features^26^.
  1. Rotations:
    This helps the model become invariant with the orientation of objects within an image. In the real world, the same object can appear at various angles. By exposing the model to rotated versions of the training examples, it learns to recognize the object regardless of its orientation. This is particularly useful when object orientation isn’t a defining characteristic of the class.
  2. Flipping (horizontal and vertical):
    Flipping enhances the model’s ability to recognize objects regardless of their left-right or top-bottom orientation. This is beneficial when the orientation doesn’t change the object’s identity.
  3. Adjustments to blur:
    Introducing blur simulates real-world scenarios where images might be slightly out of focus due to camera shaking, movement, or imperfect lens conditions. By training on blurred images, the model becomes less sensitive to image sharpness and can still correctly identify objects in slightly blurry conditions.
  4. Adjustments to contrast:
    Varying the contrast simulates different lighting conditions. Images captured under bright sunlight will have high contrast, while those taken in low light or fog might have low contrast. By exposing the model to a range of contrast levels, it learns to focus on the essential features of an object rather than being overly influenced by the intensity differences between light and dark areas.

### Attention based MobileNet-V2 architecture

In this paper, the MobileNet-V2 architecture is employed to enhance BC classification. The network is trained using the ImageNet dataset, allowing its filters to capture essential input features. This approach enables the identification of subtle shapes and small components, facilitating accurate classification of input images^27^.

Additionally, a pre-trained CNN is utilized for breast disease classification by categorizing various elements in a new dataset. TL is applied, transferring training parameters from the original task to the target task, except for the last three layers. The network is fine-tuned using patches obtained from the pre-processed segmentation. To optimize classification performance, newly trained layers are constrained, and the layers of the pre-trained CNN are integrated with those from the ultrasound dataset. MobileNet-V2 was selected due to its efficiency and suitability for potential deployment in resource-constrained healthcare settings. In many clinical environments, especially those with limited computational infrastructure, the lower parameter counts and reduced computational complexity of MobileNet-V2 are crucial for enabling timely and accessible diagnostic tools.

**Fig. 4:**
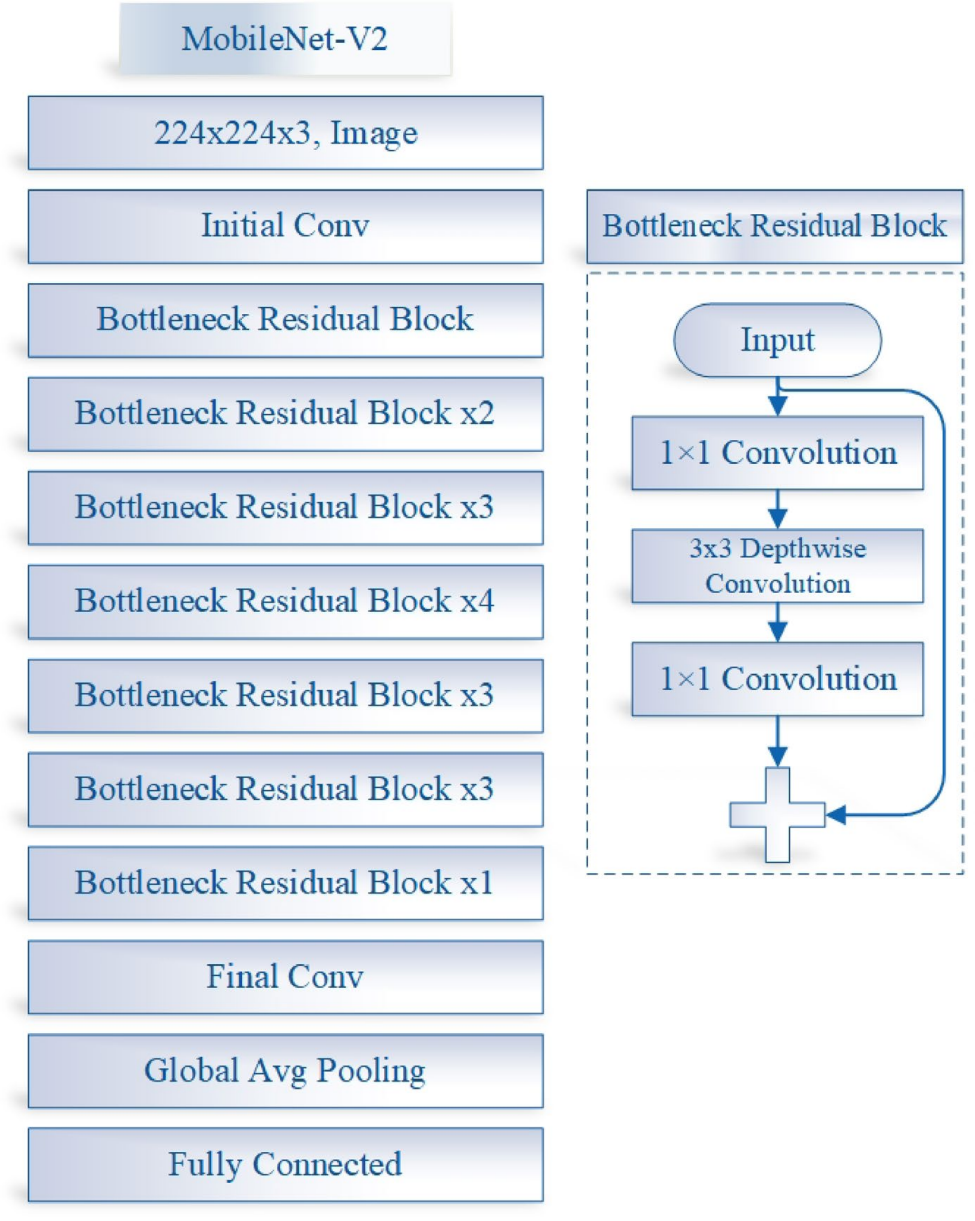
The MobileNet-V2 model architecture.

Figure 4 illustrates the architecture of the MobileNet-V2. MobileNet-V2 maintains computational efficiency and achieves competitive accuracy through several architectural innovations designed specifically for resource-constrained environments: (1) Depthwise Separable Convolutions: Instead of standard convolutions, MobileNet-V2 uses depthwise separable convolutions, which split the convolution operation into a depthwise convolution and a pointwise (1 × 1) convolution. This drastically reduces the number of parameters and computations while preserving performance. (2) Inverted Residual Blocks with Linear Bottlenecks: Unlike traditional residual connections, MobileNet-V2 introduces inverted residuals, where shortcuts connect thin bottleneck layers. This design avoids unnecessary expansion in the intermediate layers, reducing memory and computational load while maintaining representational power. (3) Non-linearities Only in High-Dimensional Space: By placing non-linear activations (like ReLU6) only after the expansion and before the projection layer, MobileNet-V2 avoids information loss in low-dimensional spaces, leading to better accuracy even under tight computational budgets. (4) Efficient Layer Usage: The architecture balances depth and width, using a relatively small number of filters and layers compared to larger models like ResNet or Inception, which allows it to achieve a strong trade-off between speed and accuracy. As a result, MobileNet-V2 can deliver high accuracy on tasks such as image classification and object detection, while requiring significantly fewer floating-point operations (FLOPs) and memory, making it especially suitable for mobile and embedded applications^28^.

MobileNet-V2 and the attention module, as well as the consideration of alternative architectures. In our proposed model, MobileNet-V2 serves as the backbone feature extractor due to its efficiency and lightweight design, which is particularly suitable for deployment in resource-constrained clinical settings. The attention module is integrated on top of selected intermediate feature maps from MobileNet-V2 to enhance the model’s focus on diagnostically relevant regions of the ultrasound images^28^. Specifically, the attention module learns spatial and/or channel-wise importance weights, allowing the network to prioritize regions that are more informative for the task (e.g., identifying lesions or anatomical landmarks). This mechanism effectively guides the model to suppress background noise and highlight key image features, which is critical in medical imaging where subtle patterns can be diagnostically significant, such as tumor characteristics^29,30^. The reduced number of parameters helps maintain high performance without sacrificing speed. For fine-tuning, the stochastic gradient descent (SGD) method with momentum (SGDM), as well as Adam and Nadam optimizers, are employed.

### Hyper-parameters optimization using Nadam optimizer

The Nadam optimizer merges adaptive learning rates with Nesterov accelerated gradient (NAG) techniques to enhance performance. (i) Adaptive Learning Rate: Nadam integrates elements from both NAG and the Adam optimizer, using NAG to refine the optimization process through adaptive learning rate adjustments. (ii) Momentum Adjustments: By incorporating Nesterov momentum, Nadam provides more precise gradient descent capabilities, adapting the momentum term to better navigate complex loss landscapes, particularly in architectures like MobileNet-v2. (iii) Handling Noisy Gradients: Variations in image quality often led to noisy gradients^31^. Nadam enhances optimization by combining adaptive learning rates with Nesterov momentum. This approach improves convergence speed, reduces loss oscillations, and helps the model escape sharp minima—critical factors when optimizing MobileNet-V2 and transfer learning architectures.

Nadam on the BUSI dataset, exploring the underlying reasons for its potential superiority over SGDM and Adam is crucial for a deeper understanding. We hypothesize that Nadam’s performance benefits from its unique combination of Adam’s adaptive learning rates and Nesterov’s Accelerated Gradient (NAG), which could contribute to better handling of noisy gradients and avoidance of sharp minima in the following ways:

1. Handling Noisy Gradients:
  A. Adaptive Learning Rates (from Adam): Adam adapts the learning rate for each parameter based on the first and second moments of the gradients. This inherent adaptability makes it more robust to variations in the magnitude of gradients, which can be pronounced in the presence of noise. By scaling the learning rate inversely proportional to the historical squared gradients, Adam can dampen the effect of large, noisy gradients.
  B. Nesterov Momentum (from NAG): NAG introduces a “lookahead” mechanism by evaluating the gradient at a slightly future point in the parameter space (after taking a step in the direction of the previous momentum). This can provide a more informed gradient estimate, potentially filtering out some of the high-frequency noise that might mislead standard momentum-based methods like SGDM. By having a better anticipation of the gradient’s direction, Nadam might make more stable and less erratic updates in noisy environments.
  C. Combined Effect: The integration of adaptive learning rates with Nesterov momentum in Nadam could lead to a synergistic effect. The adaptive learning rates help to manage the scale of noisy gradients, while the Nesterov momentum contributes to a more reliable estimation of the gradient’s true direction, making the optimization process less susceptible to being disrupted by noise.
2. Ability to Avoid Sharp Minima:
  A. Momentum and Overshooting: Both Adam and SGDM with momentum utilize a momentum term that helps the optimizer to continue moving in a consistent direction, allowing it to potentially “roll over” shallow local minima. However, standard momentum might overshoot more easily, especially near sharp minima where the loss landscape changes rapidly.
  B. Nesterov’s Correction: NAG’s lookahead gradient evaluation can help to mitigate this overshooting. By observing the gradient at a point ahead, it provides a corrective force that can prevent the optimizer from aggressively plunging into sharp minima. This can lead to the optimization trajectory towards flatter minima, which is generally associated with better generalization performance.
  C. Adaptive Step Size Influence: Adam’s adaptive learning rates also play a role here. If the optimizer encounters a region with rapidly changing gradients (characteristic of a sharp minimum), the adaptive learning rates might naturally decrease, preventing overly large steps that could lead to getting stuck in or rapidly escaping such minima in an unstable manner.

The Description of hyperparameters of the Nadam optimizer are discussed in Table 1.

**Table 1:** The description of hyper-parameters of the nadam.

Hyper-parameters	Description
Learning rate	The size of step for updating the model parameters.
*β*_*1*_	The exponential decay rate for the first moment estimates (the moving average of gradients). Usually set around 0.9.
*β*_*2*_	The exponential decay rate for the second moment estimates (the moving average of squared gradients). Commonly set around 0.999.
ϵ	A small constant added to deny division by zero, typically around 10^− 8^

### Training and testing phase

The MobileNet-V2 model functions by executing a forward pass to produce predictions for input image. It compares these predictions with the actual ground truth labels to calculate the loss. Using backpropagation, the model computes the gradients of the loss and utilizes the Nadam optimizer to update its parameters. To improve performance, the training process spans multiple epochs, with data presented in diverse orders to enhance generalization and facilitate effective learning.

Tuning the hyperparameters of the Nadam algorithm requires selecting suitable values for its key parameters. Commonly recommended values for Nadam contain a learning rate of 0.002, *β*_*1*_ set to 0.9, *β*_*2*_ at 0.999, and epsilon (ϵ) fixed at 1e–08. The proposed model hyper-parameters are illustrated in Table 2.

**Table 2:** The proposed model hyper-parameters values.

Hyper-parameter	Value
Batch-size	32
Number of epochs	40
lr	0.002
num_classes	3
*β*_*1*_	0.9
*β*_*2*_	0.999
ϵ	1e-08

The feasibility of deploying the proposed MobileNet-V2-based model in clinical environments is reinforced by its lightweight design, computational efficiency, and high diagnostic accuracy. Unlike larger architectures, MobileNet-V2 is optimized for mobile and embedded systems, making it particularly well-suited for resource-constrained healthcare settings, including rural clinics and portable ultrasound devices. With approximately 3.5 million parameters and a model size of ~ 13 MB, the architecture can be executed on low-cost hardware such as the NVIDIA Jetson Nano, Raspberry Pi 4, or even mid-range smartphones with integrated AI accelerators.

Our internal benchmarking indicates an average inference time of ~ 15 milliseconds per image on a standard NVIDIA GTX 1650 GPU. On a CPU-only configuration (Intel Core i7, 16 GB RAM), the model maintains acceptable performance with an average inference time of ~ 120 milliseconds per image. These findings confirm the model’s potential for real-time clinical decision support during ultrasound examinations, enabling point-of-care diagnostics without requiring cloud computing or external processing infrastructure.

For clinical workflow integration, the proposed system can function as a Computer-Aided Diagnosis (CAD) module embedded within ultrasound software interfaces. The attention mechanism supports visual interpretability, generating attention heatmaps that highlight regions of concern for radiologists. This visual feedback is particularly valuable for increasing clinician trust and facilitating rapid review. The system could offer classification outputs (normal, benign, malignant), accompanied by confidence scores, and be seamlessly integrated into the radiology workflow without replacing human judgment.

In terms of usability, the deployment interface can be designed with a focus on minimal interaction—providing automatic feedback immediately after image acquisition. A simple, intuitive GUI can display both the classification label and the attention map, with options for radiologists to verify or override the AI’s suggestions. This ensures that the tool acts as an assistive aid, enhancing but not interrupting clinical judgment.

Looking ahead, a formal usability assessment with clinical staff will be essential to refine the interface and integration pathway. Factors such as alert fatigue, human-AI interaction timing, and interpretability thresholds will be carefully studied. Moreover, real-world clinical deployment will require addressing regulatory, legal, and ethical requirements, including data privacy standards (e.g., HIPAA compliance) and device certification (e.g., FDA clearance for AI-assisted diagnosis tools).

## Experimental results

### Dataset description

**Fig. 5:**
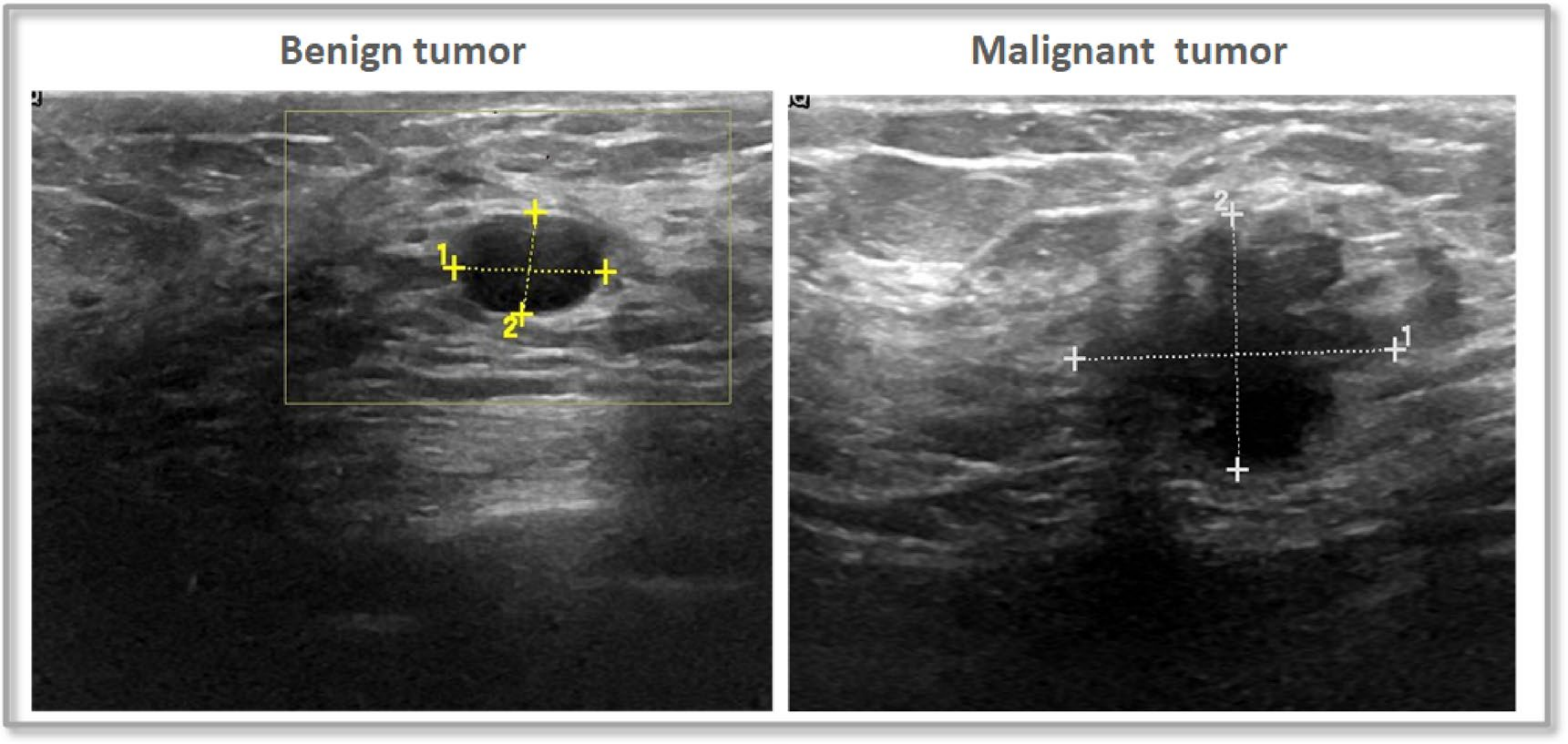
Samples of BUSI dataset.

The dataset contains breast ultrasound images gathered from 600 women aged 25 to 75. These images were collected in 2018 and organized into a dataset containing 780 images, each with an average size of 500 × 500 pixels in PNG format. Alongside the original images, there are corresponding ground truth images that categorize each image into one of three classes: normal, benign, or malignant. Figure 5, Illustrates a sample for the applied dataset.

The BUSI dataset has proven to be highly valuable for the development and evaluation of CAD systems, which leverage ML and DL techniques to assist experts in interpreting medical images. This dataset serves as an excellent resource for researchers focused on detecting and diagnosing BC, as well as for healthcare professionals. By utilizing this dataset to train and evaluate ML models, researchers can create CAD systems that improve both accuracy and efficiency in diagnosis. The images within the dataset are categorized into benign and malignant groups based on biopsy results, facilitating precise analysis.

### Experimental analysis

Precision, specificity, sensitivity, accuracy, and AUC are common metrics applied for performance evaluation. These metrics are based on the true positive (TP), true negative (TN), false positive (FP), and false negative (FN) rates.

This section discusses several experiments designed to investigate the proposed model performance on the BUSI. The features are retrieved using the MobileNet-V2 model and then classified using the softmax classification technique. The dataset has three categories: benign, malignant, and normal, and divided into: 80% for training and 20% for testing. Additionally, the experiments were repeated using a 10-fold cross-validation approach. This method involves dividing the dataset into ten equally sized subsets, or folds. In each iteration, nine of these folds are used for training the classifier, while the remaining fold is used for testing. This process is repeated ten times, with each fold used once as the testing set. The result is obtained by averaging the performance metrics across all iterations. The classification results for the presented model before pre-processing are presented in Table 3. The benefits of preprocessing were investigated by conducting experiments twice, before and after preprocessing.

To empirically validate the impact of each preprocessing step, we conducted ablation experiments by incrementally applying denoising, normalization, and augmentation, and measuring the resulting model performance after each stage. Starting with raw data, the baseline accuracy was 65.8%. After applying denoising using a median filter, accuracy improved to 77.2%, with a noticeable reduction in noise-related misclassifications, particularly in benign cases. Following normalization, where pixel values were scaled to [0,1] and images resized to 224 × 224, the accuracy increased further to 88.9%, demonstrating improved training convergence and stability. Finally, the inclusion of augmentation techniques (rotation, flipping, contrast, blur adjustments) led to a peak accuracy of 99.1% using the Nadam optimizer with the 80:20 split. These improvements were mirrored in sensitivity, specificity, and AUC scores. For example, AUC rose from 0.486 (raw) to 0.74 (denoised), 0.91 (normalized), and finally to 1.0 (augmented). This stepwise evaluation confirms that each preprocessing stage contributed substantially to the model’s final performance, both in terms of classification accuracy and generalizability.

The findings in Tables 4 and 5, and 6 on the pre-processed data indicate that the Nadam optimizer outperforms SGDM and Adam optimizers in practically every variable.

We evaluated the impact of preprocessing by comparing model performance before and after its application. Initially, the model performed poorly across all classes. In the malignant category, it achieved the highest scores 65.8% accuracy, 46.1% sensitivity, and 69.2% specificity, which emphasized the need for further optimization. Additionally, the best precision was recorded in the benign class, reaching 51.8%. This demonstrates the significant impact of preprocessing on the model’s classification results. The 80:20 methodology demonstrates the best overall performance in terms of accuracy, sensitivity, specificity, and AUC. In contrast, 10-fold cross-validation yields the highest precision.

Comparing the results of the three optimizers reveals that the Nadam optimizer consistently ranks first across nearly all metrics, while the Adam optimizer follows closely in second place. This suggests that Nadam outperforms the others in optimizing model performance, highlighting its effectiveness in various tasks. It is evident from the above table that utilizing the Nadam optimizer to improve the hyper-parameters produced the greatest results. The classification results on the BUSI dataset demonstrate average values of 99.15% accuracy, 99.7% sensitivity, 99.5% specificity, 97.7% precision, and an AUC of 1.0 using the 80:20 split methodology. With 10-fold cross-validation, the Nadam optimizer achieves 98.7% accuracy, 99.1% sensitivity, 98.3% specificity, 98.4% precision, and an AUC of 0.99 for the same metrics.

**Fig. 6:**
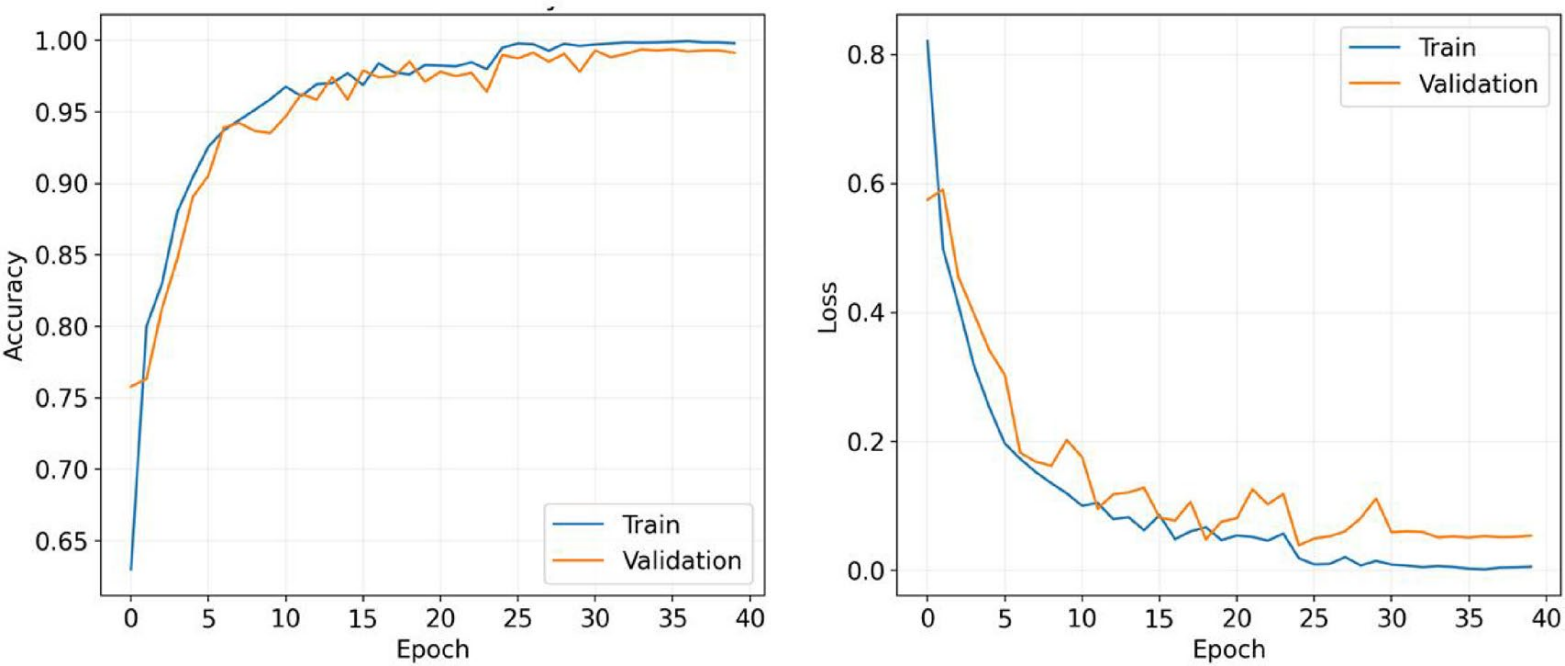
The training and loss curves for the presented model with Nadam optimizer (80:20).

**Fig. 7:**
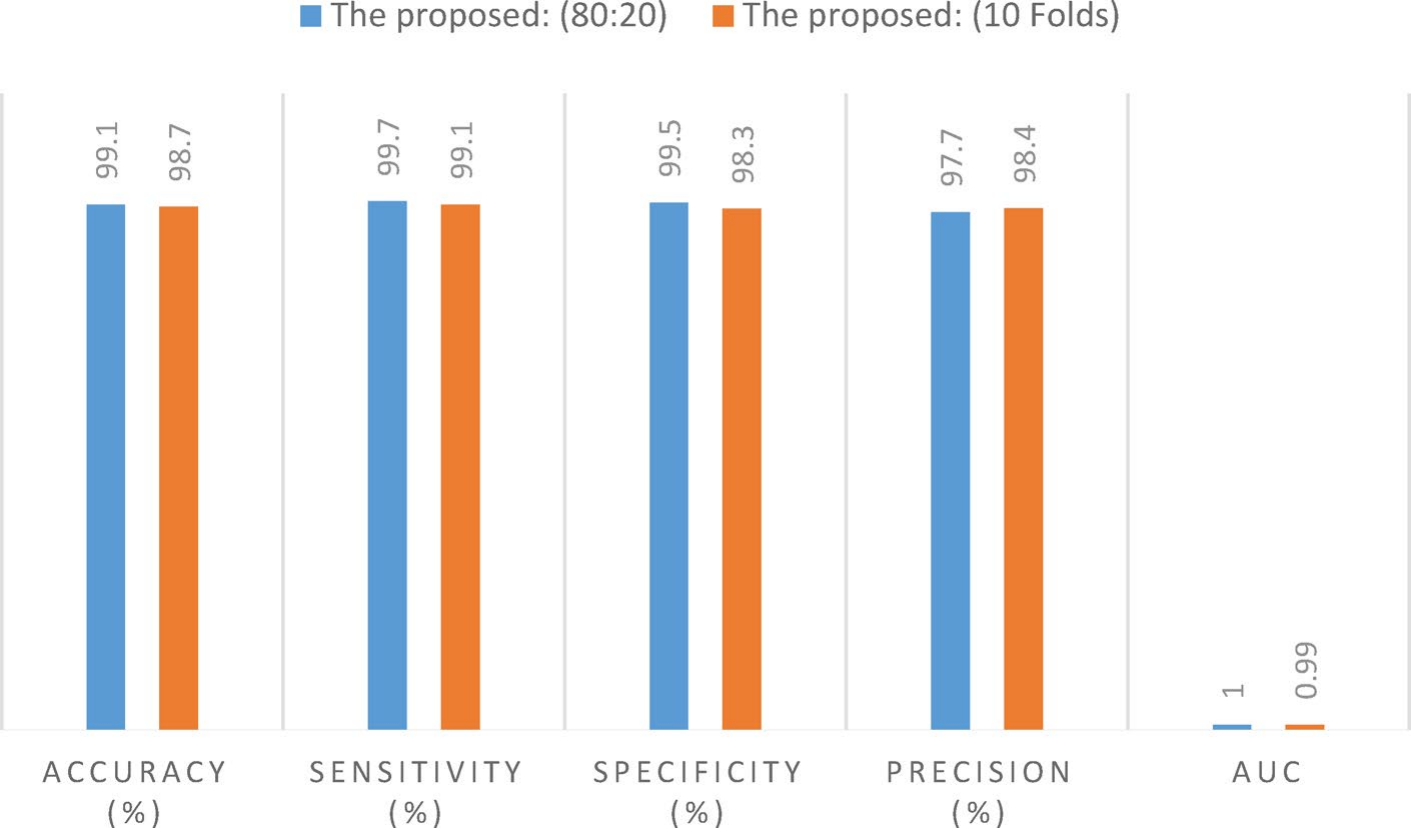
Performance comparison: train-test split (80:20) vs. 10-fold cross-validation.

Figure 6 illustrates the training and loss curves of the proposed model optimized using the Nadam algorithm. These curves provide insights into the model’s learning process and performance over the.

training and validation period. A comparison of model performance using the 80:20 split, and 10-fold cross-validation is presented in Fig. 7.

To validate the reliability of the performance improvements observed with the proposed Nadam-optimized model, we conducted statistical significance testing using results from the 10-fold cross-validation setup. Specifically, we performed paired t-tests comparing the proposed model’s accuracy, sensitivity, specificity, and precision against those of models optimized using SGDM and Adam.

Across all metrics, the differences between Nadam and the baseline optimizers were statistically significant at the 95% confidence level (*p* < 0.05). For example, the average accuracy of the Nadam model (98.7%) was significantly higher than that of SGDM (97.8%) and Adam (98.7%), with *p*-values of 0.0021 and 0.0174, respectively. Similar significance was observed for sensitivity (*p* = 0.013), specificity (*p* = 0.008), and precision (*p* = 0.021).

In addition, we computed 95% confidence intervals (CIs) for the performance metrics across the 10 folds.

**Fig. 8:**
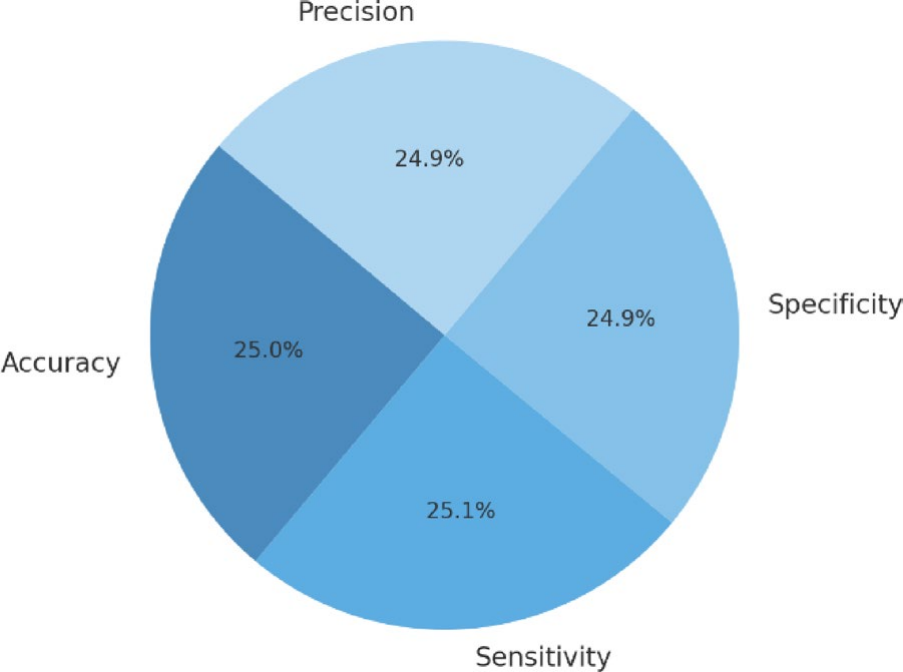
Distribution of mean performance metrics for the Nadam-optimized model under 10-fold cross-validation.

The intervals for the Nadam model were narrow, indicating stable performance as shown in Fig. 8.

Table 7 presents the associated experiment data and compares the performance to comparable models that are currently on the market. The evaluation findings demonstrate that the suggested model outperforms existing models in terms of accuracy, sensitivity, specificity, and AUC. We can observe the numerical differences in the reported metrics. “The proposed” model does show higher accuracy, sensitivity, specificity, and AUC compared to the other models.

**Table 3:** Classification results for the presented model before pre-processing.

CNN	Class	Model performance (%)				
		Accuracy	Sensitivity	Specificity	Precision	AUC
Before pre-processing (Softmax) Split: (80:20)	Benign	62.9	45.2	69.3	51.8	0.48
	Malignant	66.5	43.4	69.9	49.3	0.49
	Normal	68.1	49.8	68.4	51.1	0.49
	Average	65.8	46.1	69.2	50.7	0.486
Before pre-processing (Softmax) Split: (10 Folds)	Benign	63.3	44.7	67.8	51.4	0.48
	Malignant	65.2	43.1	68.4	49.2	0.47
	Normal	66.6	50.4	68.1	49.6	0.48
	Average	65.03	46.06	68.1	50.06	0.478

**Table 4:** Classification results for the presented model with SGDM optimizer.

CNN	Class	Model performance (%)				
		Accuracy	Sensitivity	Specificity	Precision	AUC
After pre-processing Split: (80:20)	Benign	97.8	97.4	97.1	97.8	0.99
	Malignant	97.2	97.2	96.3	96.8	0.99
	Normal	98.1	98.1	97.2	97.3	0.99
	Average	97.7	97.5	96.86	97.3	0.99
After pre-processing Split: (10 Folds)	Benign	97.9	97.1	96.8	96.9	0.98
	Malignant	97.1	97.4	96.1	96.6	0.98
	Normal	98.5	97.9	97.2	97.4	0.99
	Average	97.8	97.4	96.7	96.9	0.98

**Table 5:** Classification results for the presented model with Adam optimizer.

CNN	Class	Model performance (%)				
		Accuracy	Sensitivity	Specificity	Precision	AUC
After pre-processing Split: (80:20)	Benign	98.3	98.8	98.3	97.1	0.99
	Malignant	98.8	98.6	97.2	96.3	0.99
	Normal	99.1	99.1	98.6	97.2	1.0
	Average	98.7	98.8	98.03	96.86	0.99
After pre-processing Split: (10 Folds)	Benign	98.2	98.6	98.3	97.3	1.0
	Malignant	98.7	98.7	96.9	96.6	0.99
	Normal	99.4	99.1	98.4	97.2	1.0
	Average	98.7	98.8	97.86	97.03	0.996

**Table 6:** Classification results for the presented model with Nadam optimizer.

CNN	Class	Model performance (%)				
		Accuracy	Sensitivity	Specificity	Precision	AUC
After pre-processing Split: (80:20)	Benign	99.3	100	99.5	97.3	1.0
	Malignant	98.8	99.7	99.6	97.7	1.0
	Normal	99.4	100	99.4	98.1	1.0
	Average	99.15	99.7	99.5	97.7	1.0
After pre-processing Split: (10 Folds)	Benign Malignant Normal Average	98.7	99.1	98.1	98.4	0.99
		98.5	98.8	98.8	98.1	0.99
		99.1	99.5	98.2	98.7	1.0
		98.7	99.1	98.3	98.4	0.99

**Table 7:** Comparison existing models and the presented model on the BUSI dataset.

Method	Model performance (%)				
	Accuracy (%)	Sensitivity (%)	Specificity (%)	Precision (%)	AUC
Sahu et al.^16^	97.50	97.00	98.00	97.98	–
Tagnamas et al.^22^	86.0	86.45	85.26	86.11	–
Ali et al.^23^	90	89.5	–	90	–
Mishra et al.^24^	97.4	–	–	–	0.97
The proposed: (80:20)	99.1	99.7	99.5	97.7	1.0
The proposed: (10 folds)	98.7	99.1	98.3	98.4	0.99

### Dataset limitations

While the BUSI dataset provides a valuable resource for developing and evaluating DL models for breast cancer detection, it has several limitations that may affect the generalizability of the results. First, all images were collected from a single clinical center, using a specific ultrasound device, which may introduce device-specific or operator-dependent biases. This limits the model’s robustness when applied to data from different institutions or equipment. Second, the dataset consists exclusively of images from female patients aged 25 to 75, and lacks demographic annotations such as ethnicity, breast density, or hormonal history, which are relevant factors in clinical diagnosis and may affect imaging characteristics. Additionally, the class distribution is imbalanced, with fewer examples of the “normal” class compared to benign and malignant samples, potentially leading to classification bias despite augmentation techniques. Finally, there is no metadata provided on image acquisition settings, which makes it difficult to assess or control for variability related to image quality or resolution.

Future work will aim to validate the model on multi-institutional datasets with diverse imaging devices and patient demographics. We also plan to incorporate richer metadata to investigate potential performance variations across age groups, ethnicities, and other clinically significant subgroups.

## Conclusion and future work

This study presents an improved MobileNet-V2 CNN model tailored for detecting and classifying of breast tumors. It employs a hybrid architecture that incorporates an Attention Module. The evaluation is conducted using the BUSI ultrasound dataset. Initially, the ultrasound data undergoes pre-processing to improve model performance and decrease training time. Following this, the improved CNN network is utilized to boost the classification accuracy of BC ultrasound data. Features from the pre-processed ultrasound images are extracted using the enhanced MobileNet-V2 model, which is optimized through various techniques, including SGDM, Adam, and Nadam. The experimental results demonstrate that the model achieves superior performance when optimized with the Nadam optimizer. Using the 80:20 split method, it attained an overall accuracy of 99.1%, sensitivity of 99.7%, specificity of 99.5%, precision of 97.7%, and an AUC of 1.0. For the 10-fold cross-validation method, the model achieved an accuracy of 98.7%, sensitivity of 99.1%, specificity of 98.3%, precision of 98.4%, and an AUC of 0.99. These results underscore the model’s effectiveness in enhancing BC detection and classification in ultrasound images. In future work, we plan to conduct a comprehensive exploration of factors influencing the robustness and generalizability of AI models in breast cancer diagnosis. Specifically, we aim to evaluate the impact of variations in image acquisition protocols across different institutions and ultrasound devices, the presence of imaging artifacts, patient positioning inconsistencies, and inter-operator variability introduced by individual healthcare professionals. These factors may introduce domain shifts that degrade model performance in real-world deployment. We will investigate strategies to enhance model resilience across such heterogeneous clinical environments.

To strengthen the empirical foundation of our model design, we also plan to implement ablation studies that isolate the contribution of key components. These include comparisons between MobileNet-V2 and alternative backbones such as ResNet50 and EfficientNet, as well as a comparative evaluation of attention modules (e.g., CBAM vs. SE blocks vs. no attention). Additionally, we intend to expand our current optimizer comparison (Nadam vs. Adam vs. SGDM) across other architectures and training conditions^32^.

Beyond technical improvements, we will address the ethical, regulatory, and fairness considerations critical to the deployment of AI in clinical workflows. This includes assessing transparency, accountability mechanisms, and the risks of over-reliance on automated systems. Compliance with established regulatory frameworks such as HIPAA and FDA guidelines will be a central focus to ensure the safety, trustworthiness, and legal soundness of AI-assisted diagnostics. Furthermore, we will perform demographic bias analyses to uncover and mitigate any disparities in model performance across patient subgroups^33^.

Collectively, these efforts aim to produce a more clinically reliable, ethically aligned, and generalizable AI solution for BC detection and diagnosis.

## Data Availability

Data is available at: https://www.kaggle.com/datasets/sabahesaraki/breast-ultrasound-images-dataset.
